# Young Man with Cardiac Arrest Secondary to Undiagnosed Mediastinal Mass: A Case Report

**DOI:** 10.5811/cpcem.2020.11.49235

**Published:** 2021-01-12

**Authors:** Ian Mallett, Bjorn Watsjold, Anne K. Chipman

**Affiliations:** University of Washington, Department of Emergency Medicine, Seattle, Washington

**Keywords:** Mediastinal mass, mediastinal mass syndrome, cardiac arrest, superior vena cava syndrome

## Abstract

**Introduction:**

A 20-year-old man with a reported history of asthma presented to the emergency department in cardiac arrest presumed to be caused by respiratory failure.

**Case Report:**

The patient was discovered to have central airway obstruction and concomitant superior vena cava compression caused by a large mediastinal mass—a condition termed mediastinal mass syndrome. While the patient regained spontaneous circulation after endotracheal intubation, he was challenging to ventilate requiring escalating interventions to maintain adequate ventilation.

**Conclusion:**

We describe complications of mediastinal mass syndrome and an approach to resuscitation, including ventilator adjustments, patient repositioning, double-lumen endotracheal tubes, specialty consultation, and extracorporeal life support.

## INTRODUCTION

Masses of the mediastinum are rare. In the Framingham Heart Study, mediastinal masses were present in 0.9% patients^1^; the exact prevalence, however, is difficult to ascertain given that most are asymptomatic until they compress or invade surrounding structures. Compression of the trachea may result in complaints of shortness of breath, orthopnea, cough, or stridor. These symptoms are often exacerbated by upper respiratory tract infections, airway inflammation, or manipulation.^2^

Compression of the vena cava may result in superior vena cava (SVC) syndrome, which is characterized by intermittent swelling of the face and oropharyngeal structures, facial rubor, and/or venous congestion. Compression of pulmonary arteries may mimic massive pulmonary embolism (PE) causing acute right heart failure or circulatory collapse secondary to severely impaired pulmonary venous return, drastically reducing left ventricular preload. Collectively, this constellation of pathologies is known as mediastinal mass syndrome.^2^,^3^,^4^ Cardiopulmonary arrest has been reported secondary to compression of neck and/or mediastinal structures by large mediastinal masses causing obstructive shock, and patients can become difficult to ventilate after being given neuromuscular blockade for intubation. Providers must take care to rule out other causes of obstructive shock such as PE, cardiac tamponade, aortic stenosis, and tension pneumothorax.^4^–^6^

## CASE REPORT

A young man with a reported history of asthma presented to the emergency department (ED) with ongoing cardiopulmonary resuscitation (CPR) after suffering from cardiac arrest on the sidewalk in front of the hospital. Family of the patient reported a two-week history of worsening dyspnea prior to his presentation.

Initial examination revealed an apneic patient with distended veins across his upper chest and neck suggestive of obstructive pathology as a potential etiology of arrest, in addition to the history of respiratory distress. Medical providers inserted one 14-gauge angiocatheter in the bilateral second intercostal spaces for chest decompression, but no rush of air was noted. The cardiac monitor showed an initial rhythm of wide-complex pulseless electrical activity. With CPR ongoing, the patient was intubated without complication via direct laryngoscopy and manually ventilated with return of spontaneous circulation (ROSC) after approximately six minutes of CPR. No adjuvant medications were given prior to ROSC as the first four minutes of CPR were provided outside of the ED, and intubation with subsequent ROSC was achieved simultaneously with successful peripheral vascular access.

No oropharyngeal or airway abnormalities were noted other than minimal edema of the epiglottis. Endotracheal tube (ETT) placement was verified by auscultation, condensation within the tube, and colorimetric carbon dioxide (CO_2_) device. Initial arterial blood gas (with reference ranges in parentheses) after intubation showed a pH of 6.97 (7.35–7.45); pCO_2_ of 104 millimeters mercury (mm Hg) (reference range 35–45 mm Hg); oxygen (pO_2_) of 44 mm Hg (75–100 mm Hg); and bicarbonate (HCO_3_) of 23 milliequivalents per liter (mEq/L) (22–26 mEq/L). After ROSC, his oxygenation was 95%. The patient was given nebulized albuterol and intravenous (IV) magnesium and methylprednisolone for presumed respiratory arrest secondary to severe asthma.

**Image 1:**
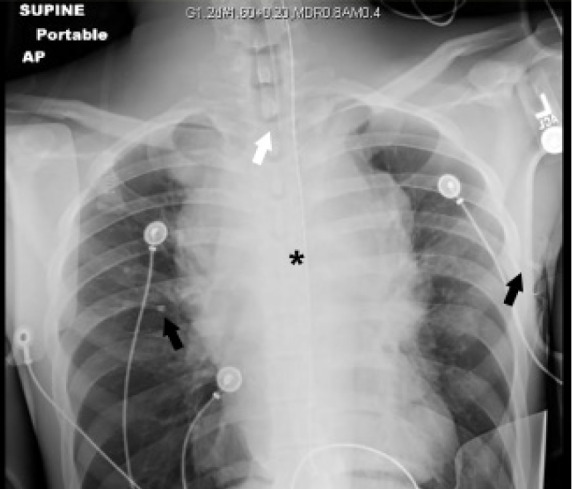
Supine anteroposterior chest radiograph. Asterisk identifies a centrally located opacity that obscures the normal mediastinal anatomy including the trachea, pulmonary vasculature and aortic contour. Black arrows denote bilateral angiocatheters used for decompression of the chest. White arrow identifies the tip of the endotracheal tube at the level of the clavicles. The lungs appear unremarkable.

After five minutes, the mechanical ventilator alarmed and then failed to ventilate, with peak inspiratory pressures (PIP) elevated to 90 centimeters of water (cm H_2_O) indicating major airway resistance. The patient’s oxygenation decreased to 82%. However, manual bag ventilation was subsequently performed without significant difficulty and with improvement of oxygenation to 95%. Point-of-care ultrasound demonstrated lung sliding bilaterally and showed a heterogeneous mass overlying the heart and obscuring parasternal views. No pericardial effusion was noted. An electrocardiogram demonstrated sinus tachycardia without obvious findings to suggest pericarditis or acute ischemia. A portable chest radiograph was obtained, which showed the ETT in appropriate position and revealed a large, central opacity obscuring the normal airway anatomy and cardiac silhouette (Image 1).

**Image 2:**
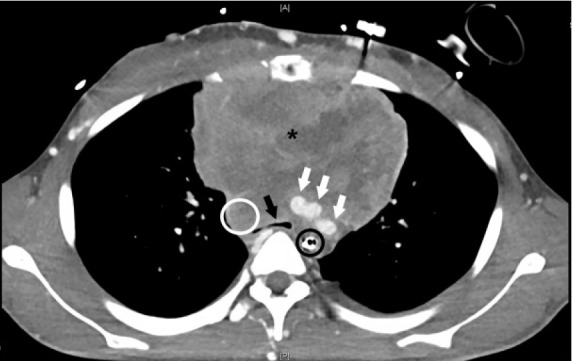
Axial computed tomography with contrast image of the chest. Contrast within the vessels appears white. A heterogeneously enhancing mass is seen occupying the anterior mediastinum (asterisk). There is significant compression of the trachea (black arrow) at the level of the carina, which is smaller in diameter than the adjacent orogastric tube (black circle). White arrows indicate brachiocephalic, left carotid, and left subclavian vessels just superior to their origins from the aortic arch. The superior vena cava (which should be in the vicinity of the white circle) is conspicuously absent.

Providers recognized that a large mediastinal mass might be compressing the airway, accounting for the high inspiratory pressures. The ETT was advanced approximately four cm (as far as possible without meeting resistance) attempting to bypass the obstruction; the ventilator was reconnected after manual bag ventilation and ventilated the patient appropriately with the original setting of positive end-expiratory pressure (PEEP) 5 cm H_2_0. Computed tomography (CT) demonstrated a large mass in the anterior mediastinum, with almost complete obliteration of the SVC with significant collateralization, as well as severe stenosis of the airways at the level of the lower one-third of the trachea, carina, and main-stem bronchi (Image 2).

While the patient was undergoing CT the ventilator again failed secondary to increased PIPs, and manual bagging was reinitiated. PEEP was increased to 10 cm H_2_O, and providers raised the patient to a semi-recumbent position, which allowed the ventilator to oxygenate with PIPs in the 40s. Prior to intensive care unit admission, a cooling catheter was placed in the common femoral vein. Repeat blood gas showed improvement with a pH of 7.33 and downtrending lactate.

**CPC-EM Capsule**

What do we already know about this clinical entity?

Mediastinal masses are rare and can become large enough to cause compression of the trachea or heart. This is called “mediastinal mass syndrome (MMS).”

What makes this presentation of disease reportable?

MMS led to cardiac arrest in a young, otherwise healthy patient who was difficult to ventilate secondary to tracheal compression.

What is the major learning point?

Simple steps such as patient repositioning and increasing positive end-expiratory pressure may be life saving in cases of MMS.

How might this improve emergency medicine practice?

Providers should be aware of MMS, the steps to correct resulting central airway obstruction and also consider extracorporeal membrane oxygenation.

The patient regained consciousness after cooling and rewarming but demonstrated diffuse myoclonus affecting the diaphragm, preventing extubation, and ultimately required tracheostomy.

Tissue sampling of the mass revealed a large B-cell lymphoma, and chemotherapy was started. He subsequently developed massive hemorrhage from his right common carotid artery and jugular vein due to malignant erosion into these vessels. He suffered a subsequent arrest and was resuscitated in the operating room with ligation of the bleeding vessels but had diffuse brain injury on magnetic resonance imaging (MRI). His family agreed that further treatment was unlikely to lead to meaningful recovery, and he died on hospital day 52 after care was withdrawn.

## DISCUSSION

In regard to anterior mediastinal masses, the most common etiologies are taught as the “4 Ts”: thymoma, thyroid, teratoma, and “terrible” lymphoma. We describe cardiopulmonary arrest in an otherwise healthy young man due to an undiagnosed mediastinal mass. Although the patient regained spontaneous circulation after his airway was secured by endotracheal intubation, he remained difficult to ventilate due to lower airway compression. Mediastinal mass syndrome can lead to arrest due to compression or obstruction of critical structures, including airway obstruction, as in our patient. Circulatory obstruction leading to obstructive shock is possible due to massive PE, pericardial effusion causing tamponade physiology, direct compression of the heart by the mass, and positional SVC syndrome causing sudden decrease in cardiac venous return.^4^ Recognizing central obstruction will allow providers to appropriately manage abnormal respiratory and circulatory physiology.

In the case of the decompensating ventilated patient, providers should first assess for complications arising from mechanical ventilation in a stepwise fashion to aid in decision-making. One popular mnemonic for this approach is “**DOPES**”: **D**islodgement/displacement of the ETT or cuff; **O**bstruction of the airway or ETT; **P**neumothorax; **E**quipment failure (of the ventilator or tubing); and “**S**tacking” (referring to auto-PEEP or “breath-stacking).”^7^ In our patient, ventilation was complicated by lower airway obstruction. Normal airway peak inspiratory pressure values are ideally less than 30 cm H_2_0, but 30–40 is considered acceptable. In our patient, PIP was markedly elevated indicating high resistance to airflow through the tracheobronchial tree, ETT or ventilator tubing which caused ventilatory failure, as opposed to elevated PIP ***and*** plateau pressures, which would have indicated a lung compliance issue. High PIP in the setting of normal plateau pressures indicates obstruction and several causes of obstructive physiology must be evaluated. These include the following: kinked ventilator tubing; twisted ETT or ETT partially occluded by blood, mucus, etc.; patient biting ETT; bronchospasm/asthma; and trachea or large bronchus *partial* occlusion by mass or mucus plugging. 8

Our patient required advancement of the ETT past the obstructed segment, PEEP, and repositioning to maintain adequate ventilation. Review of the literature indicates these and several other interventions may alleviate central airway obstruction. Repositioning the patient from supine to a semi-recumbent or upright position may both relieve compression of the central airway and increase venous return to the right heart, increasing preload.^5^,^9^ In addition, increasing PEEP has also been suggested as a means to artificially stent open the large airways and relieve obstruction while awaiting more definitive management.^10^ However, providers must be judicious with increasing the PEEP as it may further reduce venous return to the heart (especially in patients with SVC compression) and/or cause compression of the distal airways secondary to increased intrathoracic pressures.

Passage of a double lumen or wire-reinforced ETT, or placement of an endotracheal stent via bronchoscopy, can also be performed to relieve airway obstruction. Surgical or radiologic removal/debulking of the offending mass may be necessary in the subacute phase of care.^2^, ^11^,^12^ If providers are still unable to ventilate due to central airway obstruction, they may consider extracorporeal life support (ECLS). While some authors have argued against ECLS as an intervention given the prolonged time necessary to initiate this intervention and unlikely favorable outcomes due to hypoxic brain injury,^13^ other case reports have indicated that patients have survived their initial cardiac arrest to receive other potentially lifesaving interventions.^4^,^5^,^11^ It is our opinion that providers should consider ECLS in these patients and consult their institution’s ECLS specialists if available, according to their institutional protocols.

In addition to airway compression, patients with large mediastinal masses are also at high risk of SVC syndrome. The literature advocates for obtaining vascular access in the lower extremities such as a femoral central line in cases where the SVC may be severely stenosed or occluded.^3^,^5^,^14^ In such cases, the distribution and effect of IV drugs such as epinephrine or induction agents may be slowed secondary to said occlusion and limit successful resuscitation, thus making access to the inferior vena cava via the lower extremity vasculature a critical component of successful resustitation.^10^ As noted above, raising the patient to a semi-recumbent or upright position may relieve compression of the SVC by shifting the position of the mass relative to the force of gravity. It may also decrease the size of the mass by decreasing venous congestion within the mass itself. However, patients may require endovascular stenting of the SVC if they continue to experience significant symptoms due to compression, and for this reason, providers should consider consultation with cardiothoracic or vascular surgery depending on institutional protocols.^15^

## CONCLUSION

Cardiac arrest from mediastinal mass syndrome is rare and can be difficult to manage. Effective management of airway obstruction and adequate ventilation depend on stenting the airway to relieve obstruction. In the emergent setting, options are limited, and relieving airway obstruction must be accomplished with available tools, namely endotracheal intubation, patient repositioning, and positive end-expiratory pressure. Specialty consultation may be both helpful and necessary, and providers should consider ECLS in the patients who remain unstable. In patients with high suspicion for SVC syndrome providers should also consider obtaining vascular access in the lower extremities as well as potential specialist consultation for endovascular stenting of the SVC.
